# Ferulic acid via attenuation of oxidative stress and neuro-immune response utilizes antinociceptive effect in mouse model of formalin test

**DOI:** 10.1016/j.ibneur.2023.12.001

**Published:** 2023-12-14

**Authors:** Shima Balali-Dehkordi, Saeid Habibian-Dehkordi, Hossein Amini-Khoei, Rahil Mohajerian

**Affiliations:** a Department of Basic Sciences, Veterinary Faculty, Shahrekord University, Shahrekord, Iran; b Medical Plants Research Center, Basic Health Sciences Institute, Shahrekord University of Medical Sciences, Shahrekord, Iran; c Department of Basic Sciences, Veterinary Faculty, Shahid Chamran University of Ahvaz, Ahvaz, Iran

**Keywords:** Ferulic acid, Pain, Formalin test, Oxidative stress, Neuroinflammation

## Abstract

**Introduction:**

Plenty evidences suggests that neuroinflammation and oxidative stress augmented the neural sensitivity specifying that neuro-immune response is involved in the pathophysiology of pain. Ferulic acid (FA), a natural antioxidant found in various fruits, has various pharmacological properties. The purpose of the current study was to assess the antinociceptive effect of FA in a mouse model of formalin test with focus on its anti-neuroinflammatory and antioxidative stress effects.

**Methods:**

The injection of FA (40 mg/kg), piroxicam (2 mg/kg), and saline (0.9% NaCl) (1 ml/kg) was done intraperitoneally and after one hour, formalin injected into the plantar surface of the hind paw of mice. Then pain behavior was documented during 60 min. Then mice were euthanized and prefrontal cortex (PFC) samples were taken. Malondialdehyde (MDA) level, antioxidant capacity and expression of inflammatory genes, counting tumor necrosis factor (TNF-) and interleukine 1 (IL-1) evaluated in the PFC.

**Results:**

exhibited that FA declined the pain behavior following injection of formalin. Besides, FA significantly diminished the MDA level and increased the antioxidant capacity in the PFC. We revealed that FA diminished the expression of TNF-α and IL-1β genes in the PFC.

**Conclusion:**

We conclude that FA exerted antinociceptive effects in the formalin test in mice, at least partially, by reducing oxidative stress and neuroimmune response in the PFC.

## Introduction

Pain is a defense response which alert subjects about an injury or damage of organs (Polomano et al., 2016). There are several mediators involved in the pathophysiology of pain including neurotransmitters and inflammatory cytokines (Butrick, 2016). In this regards, previous studies have shown that cytokines accelerated the pain response as well as increased the sensitivity to pain in the brain (Zhang and An, 2007). Inflammatory cytokines has crucial role in sensitization of nociceptor sensory neurons and amplification of pain response (Pinho-Ribeiro et al., 2017). There are two type of pain including acute and chronic pain (Bliss et al., 2016). In the formalin test, an inflammatory response considered as chronic pain behavior (Fan et al., 2018). Injection of formalin create two phases of pain including early phase (acute pain) during first 5 min and the late phase (inflammatory pain) during 15–60 min after injection (Hacimuftuoglu et al., 2006). Previous studies have demonstrated that agents which mitigated inflammatory cytokines, effectively reduced inflammatory pain responses in the formalin test (Pereira et al., 2009, Dray and Dickenson, 1991). It has been shown that there is a crosslink between inflammatory responses and chronic pain (Deforest et al., 2018). Evidences have shown that chronic pain induced inflammatory response in the brain parts involved in pain processing (Deforest et al., 2018, Bliss et al., 2016). The formalin test is a valid trial to evaluate chronic inflammatory pain in rodent (Bagdas et al., 2015).

Previous studies have determined that oxidative stress is involved in the pathophysiology of pain (Fusco et al., 2019). In this context, oxidative and nitrosative stresses accelerated the pain response and led to a hyperalgesia state (Tu et al., 2020). There are studies reported that oxidative stress in parts of the brain relevant to pain behavior such as PFC accelerate and potentiate the pain response (Sagalajev et al., 2018, Tanwar et al., 2020). In this regards, arising body of studies have shown that inhibition of oxidative stress could significantly attenuated the pain response (Zhao et al., 2020, Gouveia et al., 2018). Increase in the malondialdehyde (MDA) levels in the central nervous system is associated with promoting pain behavior (Herzberg et al., 2019). It has been determined that oxidative/antioxidative imbalance as decrease in the antioxidant capacity is linked with chronic pain response (Rondanelli et al., 2018, Zhang et al., 2019).

Ferulic acid (FA) ([E]−3-[4-hydroxy-3-methoxy-phenyl] prop-2-enoic acid) is a phenolic compound which found in certain fruits (Zduńska et al., 2018). Preceding studies have revealed that FA exerted antioxidant and anti-inflammatory effects (Ghosh et al., 2017). It has been demonstrated that FA revealed various pharmacological activities counting anti-inflammatory, anti-apoptotic, anti-carcinogenic, anti-diabetic, and neuroprotective effects (Ghosh et al., 2017, Hussain et al., 2017, Vashisth et al., 2015). So, considering of (1) involvement of inflammatory response and oxidative stress in the pathophysiology of pain and, (2) pharmacological properties of FA, the goal of this study was to assess FA's antinociceptive effects in the formalin test in mice, focusing on its anti-inflammatory and anti-oxidative stress properties.

## Materials and methods

### Animals and study design

Twenty-four male Naval Medical Research Institute (NMRI) mice with weight of 20–30 g were bought from the Shahrekord University of Medical Sciences, Shahrekord, Iran. Mice were kept in a standard laboratory environment (temperature of 22 ± 2 °C and 12/12 h light/dark cycle) with free access to commercial pellets and tap water. Mice were randomly divided into three groups (each group contain 8 mice) as follows: group 1 as the control group treated with saline (0.9% NaCl) (1 ml/kg), group 2 received FA at dose of 40 mg/kg (Lorigooini et al., 2020a) and group 3 as positive control group treated with piroxicam at dose of 2 mg/kg (Dellal et al., 2018). All drugs were acutely injected via intraperitoneal (i.p.) route. One hour after, formalin injected into the plantar surface of the hind paw. Then animals were euthanized below profound anesthesia using diethyl ether, PFC harvested and the total antioxidant capacity, MDA level and the gene expression of IL-1β and TNF-α were measured in the PFC.

## Formalin test method

In the formalin trial using a 27-gauge needle, 20 microliter of 2.5% formalin was injected subcutaneously into the plantar area of the left posterior paw. Immediately after the injection, the animal's behavior was recorded every 15 s for 60 min (Dehkordi et al., 2017, Dehkordi et al., 2019). Formalin test consists of first phase (0–5 min) and late (inflammatory) phase (15–60 min) in which the animal shows painful behaviors. These phases are separated with a quiet phase named interphase, in which the nociceptive responses are decreased or wholly disappeared. Mean nociceptive scores for the first phase (first 5 min), inter phase (5–15 min) and inflammatory (late) phase (15–60 min) were documented (Dubuisson and Dennis, 1977).

## Measurement of the malondialdehyde (MDA)

The concentration of MDA in the PFC samples was measured as described previously (Salimian et al., 2022, Rao et al., 2021). Mice were euthanized under anesthesia using diethyl ether, and the PFC was dissected on the ice-cold surface and directly placed into liquid nitrogen. PFC homogenates were prepared. Then 1 ml of the PFC homogenate was incubated at 37 ± 1 °C for 60 min. Tetrachloroacetic acid and thiobarbituric acid were added to it, then centrifuged at 2000 g for 15 min. Then, the supernatant solution was placed in a warm water bath and its absorbance was measured at 535 nm (Omidi-Ardali et al., 2019).

## Measurement of antioxidant capacity

By ferric reducing/antioxidant power (FRAP) assay, the antioxidant capacity was determined in the PFC samples. This technique measure ability of the samples to reduce ferric (Fe^3+^) ions to ferric (Fe^2+^) in the attendance of TPTZ (2,4,6-Tris(pyridyl)-s-triazine). In this method, the reaction of (Fe^2+^) with TPTZ reagent produces a blue complex. Absorbance was measured by a spectrophotometer in 593 nm (Salimian et al., 2022).

## RT-PCR for evaluation of gene expression of inflammatory markers

The gene expression of inflammatory cytokines including IL-1β and TNF-α was investigated by Real time-PCR method in the PFC samples. The reaction was done in triplicate for every gene and was reiterated double. The requisite specific primers were planned using Primer 3 software version 0.4.0 (http://frodo.wi.mit.edu). The H2afz gene was used as a normalizer to compare the expression of favorite genes (Ghasemi-Dehnoo et al., 2023). Lastly, the data gained from Real-time RT-PCR were considered using the PFAFFL formula. Table 1 presents primer sequences.

**Table 1:** primer sequences.

Primer	Forward sequence	Reverse sequence
H2AFZ	TCATCGACACCTGAAATCTAGGA	AGGGGTGATACGCTTTACCTTTA
TNF-α	CTGAACTTCGGGGTGATCGG	GGCTTGTCACTCGAATTTTGAGA
IL-1β	GAAATGCCACCTTTTGACAGTG	TGGATGCTCTCATCAGGACAG

## Statistical examination

Evaluation among experimental groups was done by Two-way repeated measure ANOVA followed by tukey’s post-hoc test for pain behavior and One-way ANOVA followed by tukey’s post-hoc test for biochemical and molecular findings. Data was presented as Mean ± SEM. Graph Pad Prism software (version 7) used for data analysis. P-value < 0.05 was considered as statistically significant.

## Results

### FA decreased the nociceptive scores following formalin test

**Fig. 1:**
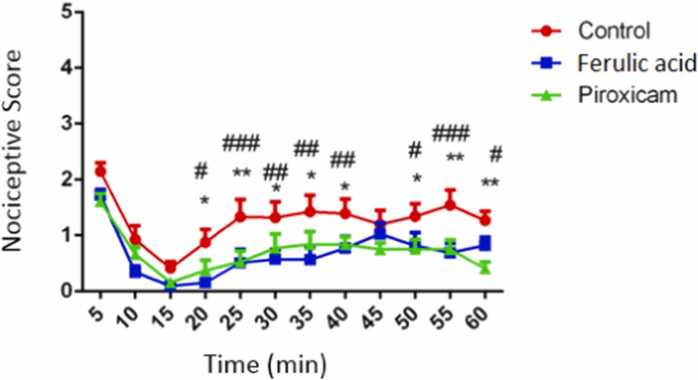
Nociceptive scores following formalin test among experimental groups. Data are obtainable as mean ± S.E.M from eight animals and were analysed using Two-way ANOVA followed by tukey’s post hoc test. *p < 0.05 and **p < 0.01 related piroxicam-received group with the control mice, #p < 0.05, ##p < 0.01 and ###p < 0.001 related FA -received group with the control mice.

The Mean ± SEM of nociceptive scores in experimental groups illustrated in Fig. 1 and Table 2. Administration of FA at 20 (p < 0.05), 25 (p < 0.001), 30) (p < 0.01), 35 (p < 0.01), 40 (p < 0.01), 50 (p < 0.05), 55 (p < 0.001) and 60 (p < 0.05) min following formalin injection significantly decreased nociceptive scores in related to the control group. Furthermore, we observed that injection of piroxicam at 20 (p < 0.05), 25 (p < 0.01), 30 (p < 0.01), 35 (p < 0.01), 40 (p < 0.01), 50 (p < 0.05), 55 (p < 0.01) and 60 (p < 0.01) min following formalin injection significantly diminished nociceptive scores in related to the control group. As Table 3 illustrates, the Mean ± SEM of nociceptive scores in the first phase in the piroxicam group significantly decreased in compared to the control group (P < 0.05). The nociceptive scores in the inter phase in the FA-received group significantly decreased in compared to the control group (P < 0.01). In the inflammatory (late) phase, results showed that in the FA-received group (P < 0.01) and piroxicam- received group (P < 0.01) nociceptive scores significantly deceased in compared to the control group.

**Table 2:** Mean ± SEM of pain scores in the experimental groups.

Time (min) Group	5	10	15	20	25	30	35	40	45	50	55	60
Control	2.15 ± 0.15	0.93 ± 0.25	0.41 ± 0.12	0.88 ± 0.23	1.34 ± 0.30	1.32 ± 0.27	1.43 ± 0.29	1.39 ± 0.26	1.20 ± 0.25	1.34 ± 0.23	1.54 ± 0.27	1.27 ± 0.16
Ferulic acid	1.72 ± 0.10	0.34 ± 0.11	0.10 ± 0.12	0.16 ± 0.09	0.51 ± 0.24	0.58 ± 0.18	0.57 ± 0.24	0.77 ± 0.21	1.03 ± 0.20	0.82 ± 0.23	0.69 ± 0.17	0.82 ± 0.15
Piroxicam	1.61 ± 0.14	0.67 ± 0.12	0.16 ± 0.08	0.37 ± 0.19	0.53 ± 0.20	0.77 ± 0.25	0.84 ± 0.22	0.84 ± 0.14	0.75 ± 0.11	0.76 ± 0.17	0.75 ± 0.17	0.41 ± 0.12

**Table 3:** nociceptive scores (mean±SEM) in different phases in the experimental groups. Data are presented as mean ± S.E.M from eight animals and were analysed using one-way ANOVA followed by Tukey’s post hoc test. *p < 0.05 and * *p < 0.01 in compared to the control mice.

Groups	First phase	Inter phase	inflammatory (late) phase
Control	2.15 ± 0.15	0.67 ± 0.17	1.27 ± 0.15
Ferulic acid	1.72 ± 0.10	0.22 ± 0.06^**^	0.66 ± 0.11^**^
Piroxicam	1.61 ± 0.14*	0.415 ± 0.08	0.67 ± 0.12^**^

## FA reduced level of MDA in the PFC

**Fig. 2:**
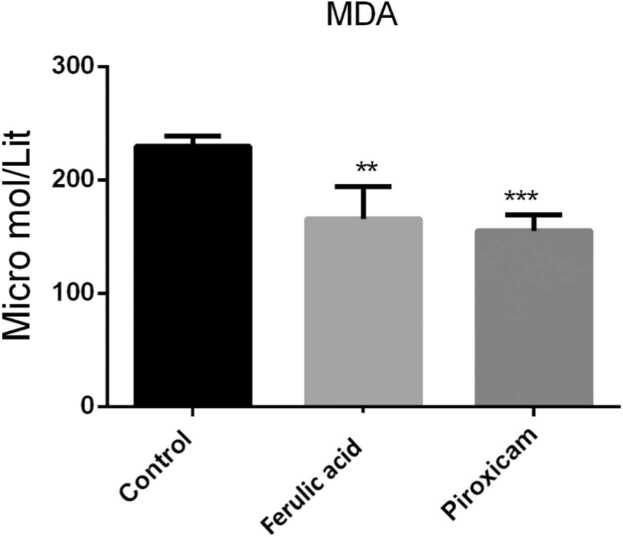
MDA level in the PFC samples among experimental groups. Data are obtainable as mean ± S.E.M from eight animals and were analysed using one-way ANOVA followed by tukey post hoc test. * *p < 0.01 and * **p < 0.001 related to the control mice.

Results demonstrated that administration of FA significantly reduced level of MDA in the PFC in compared to the control mice (P < 0.01, Fig. 2). Furthermore, administration of piroxicam significantly diminished level of MDA in the PFC in compared to the control counterpart (P < 0.001).

## FA increased antioxidant capacity in the PFC

**Fig. 3:**
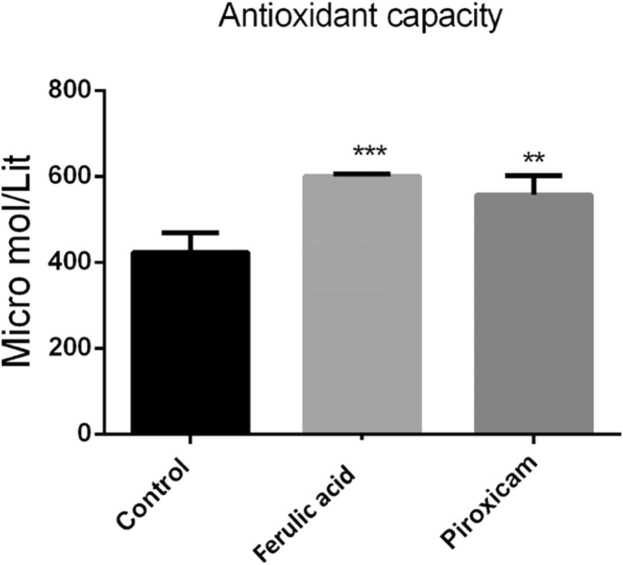
Antioxidant capacity in the PFC among experimental groups. Data are obtainable as mean ± S.E.M from eight animals and were analysed using one-way ANOVA followed by Tukey’s post hoc test. * *p < 0.01 and * **p < 0.001 related to the control mice.

Finding revealed that FA significantly augmented antioxidant capacity in the PFC when compared to the control mice (P < 0.001, Fig. 3). In addition, piroxicam significantly augmented antioxidant capacity in the PFC in compared to the control mice (P < 0.01).

FA decreased gene expression of TNFα- and IL-1β in the PFC.

**Fig. 4:**
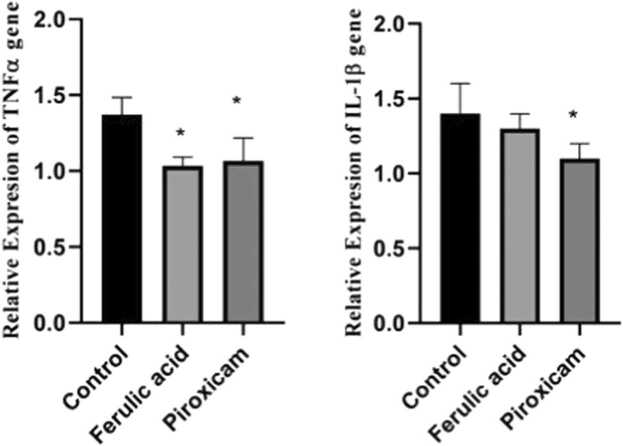
The gene expression of TNF-α and IL-1β in the PFC among experimental groups. Data are obtainable as mean ± S.E.M from eight animals and were analysed using one-way ANOVA followed by Tukey’s post hoc test. *p < 0.05 related to the control mice. Table legends.

Results demonstrated that administration of FA significantly lessened the expression of TNF-α but not IL-1β gene in the PFC in compared to the control mice (P < 0.05, Fig. 4). Furthermore, administration of piroxicam significantly reduced the expression of TNF-α and IL-1β genes in the PFC in compared to the control mice (P < 0.05).

## Discussion

The finding of the current study displayed that FA decreased the pain scores in inflammatory phase of the formalin- induced pain. We observed that FA reduced the MDA level, augmented the antioxidant capacity as well as lessened the expression of TNF-α gene in the PFC.

There are various methods to evaluate pain behavior in laboratory animals. One of these methods is formalin test. Former studies have revealed that following injection of formalin on the hind paw surface, animals showed pain behaviors (Dehkordi et al., 2019, Dehkordi et al., 2017). Injection of formalin beneath the skin of the dorsal surface of the hind paw create two phasic pain including acute and chronic (inflammatory) phases. The acute phase create throughout first 5 min afterward injection of formalin. While, inflammatory phase create from 20 to 60th min after formalin injection (Dehkordi et al., 2019). The acute phase is because of activation of nociceptors, while the chronic stage is an inflammatory response (Kumar and Vinayak, 2020, Jha et al., 2013, Allagui et al., 2022). By the way, evidences have demonestrated that attenuation of the inflammatory response led to antinociceptive effects in formalin test (Allagui et al., 2022, Gwon et al., 2021). Previous studies have reported a key role for oxidative stress in the pathophysiology of pain in formalin test (Basit et al., 2022, Kiasalari et al., 2017). In this context, earlier studies have revealed that attenuation of oxidative stress markers including MDA is involved in the analgesic effects of some agents (Forouzanfar et al., 2022, Gomes Junior et al., 2020). Furthermore, increase in the total antioxidant capacity could potentiated the antinociceptive effects of some analgesic drugs (Chakraborty et al., 2012). Antioxidants could be considered as potential analgesics in neuropathic and inflammatory pains (Khalil and Khodr, 2001, Wang et al., 2004). The PFC via its connections to other areas of the brain has a pivotal role in pain processing. It has been well-stablished that during acute and chronic pain, changes in neurotransmitters, gene expression, glial cells, and neuroinflammation in the PFC alter its structure, activity and connectivity which affect pain proccessing (Ong et al., 2019, Lorenz et al., 2003, Kummer et al., 2020). It has been determined that losses in PFC grey matter following chronic pains reverse after successful treatments(Ong et al., 2019). Chronic neuroinflammation may lead to trimming of dendritic branches, reduced synaptic contacts, lessening in pyramidal neuron output from the PFC which led to loss of antinociception (Zhang et al., 2008). It has been demonstrated that xpression of inflammatory cytokines increased in the PFC of mice with chronic pain(Gonzalez-Sepulveda et al., 2016). Abovementioned studies indicating that PFC could be consider as a target tfor pain managing.

Ferulic acid (FA) ([E]−3-[4-hydroxy-3-methoxy-phenyl] prop-2-enoic acid) is a phenolic compound commonly found in some fruits (Lorigooini et al., 2020b). Numerous pharmacological properties have been reports for FA counting anti-inflammatory, antioxidant, anticancer, anxiolytic cardioprotective, neuroprotective and antidiabetes effects (Nile et al., 2016, Zheng et al., 2019, Pandi et al., 2022, Dong and Huang, 2022). It has been determined that FA through its anti-oxidant and anti-inflammatory properties exerted nephroprotective effects in cyclosporine-induced nephrotoxicity in rats (Ghasemi-Dehnoo et al., 2023). Some studies have been reported that FA exerted antinociceptive effects (Xu et al., 2016, Xu et al., 2013), however, the detailed and complete underlying mechanisms mediating the antinociceptive effects of FA are not understood. In this study, we found that FA reduced the MDA level and augmented the antioxidant capacity in the PFC. Furthermore, we reveald that FA lessened the expression of TNF-α gene in the PFC of mice which underwent formalin injection.

## Conclusion

We concluded that, at least in part, FA exerted its antinociceptive effects in the formalin test in mice by attenuating oxidative stress and neuroinflammation in the PFC.

## Ethics

All procedures in this study were conducted in agreement with the Guide for the Care and Use of Laboratory Animals approved by the Ethics Committee of the Shahrekord University (Ethics code: IR.SKU.REC.1401.052). Every effort was made to minimize animal suffering and reduce the number of animals used.

## Funding

This study was supported by a research grant (OJRD34M1888/1400) from Shahrekord University, Shahrekord, Iran.

## CRediT authorship contribution statement

S.H-D: Performed the experiments; wrote the paper. R.M: Performed the experiments; wrote the paper. H.A-K: Analyzed and interpreted the data, performed the experiments. S. B-D: Conceived and designed the experiments; contributed reagents, materials, analysis tools or data; performed the experiments; wrote the paper.

## Consent to participate

Not applicable.

## Consent to publish

All authors reviewed and approved the manuscript.

## Declaration of Competing Interest

The authors have no conflicts of interest to declare regarding the study described in this article and the preparation of the article.

## Data Availability

No data was used for the research described in the article.
